# High-Density Genetic Mapping with Interspecific Hybrids of Two Sea Urchins, *Strongylocentrotus nudus* and *S*. *intermedius*, by RAD Sequencing

**DOI:** 10.1371/journal.pone.0138585

**Published:** 2015-09-23

**Authors:** Zunchun Zhou, Shikai Liu, Ying Dong, Shan Gao, Zhong Chen, Jingwei Jiang, Aifu Yang, Hongjuan Sun, Xiaoyan Guan, Bei Jiang, Bai Wang

**Affiliations:** 1 Liaoning Key Lab of Marine Fishery Molecular Biology, Liaoning Ocean and Fisheries Science Research Institute, Dalian, Liaoning, 116023, China; 2 The Fish Molecular Genetics and Biotechnology Laboratory, Aquatic Genomics Unit, School of Fisheries, Aquaculture and Aquatic Sciences and Program of Cell and Molecular Biosciences, Auburn University, Auburn, AL, 36849, United States of America; National Cheng-Kung University, TAIWAN

## Abstract

Sea urchins have long been used as research model organisms for developmental biology and evolutionary studies. Some of them are also important aquaculture species in East Asia. In this work, we report the construction of RAD-tag based high-density genetic maps by genotyping F_1_ interspecific hybrids derived from a crossing between a female sea urchin *Strongylocentrotus nudus* and a male *Strongylocentrotus intermedius*. With polymorphisms present in these two wild individuals, we constructed a female meiotic map containing 3,080 markers for *S*. *nudus*, and a male meiotic map for *S*. *intermedius* which contains 1,577 markers. Using the linkage maps, we were able to anchor a total of 1,591 scaffolds (495.9 Mb) accounting for 60.8% of the genome assembly of *Strongylocentrotus purpuratus*. A genome-wide scan resulted in the identification of one putative QTL for body size which spanned from 25.3 cM to 30.3 cM. This study showed the efficiency of RAD-Seq based high-density genetic map construction using F_1_ progenies for species with no prior genomic information. The genetic maps are essential for QTL mapping and are useful as framework to order and orientate contiguous scaffolds from sea urchin genome assembly. The integration of the genetic map with genome assembly would provide an unprecedented opportunity to conduct QTL analysis, comparative genomics, and population genetics studies.

## Introduction

Sea urchins have been popularly used as research model organisms to address questions in many aspects of biological sciences [1], including developmental biology, biochemistry, cell and molecular biology, as well as evolutionary biology. Some sea urchins are also important aquaculture species in East Asia, including China, Japan and Korea. The sea urchin industry has been expanded rapidly since 1990s. Two main sea urchin species, *Strongylocentrotus nudus* and *Strongylocentrotus intermedius*, are widely cultured in China. The *S*. *nudus* is a native species in China, which is mainly cultured in the northern areas, while *S*. *intermedius*, introduced from Japan in 1989, is cultured more extensively along the coast of northern China. Studies have shown that the interspecific hybrids generated by mating *S*. *nudus* with *S*. *intermedius* exhibited hybrid vigor in growth and disease resistance [2, 3]. The heterosis resulting from interspecific hybridization of other sea urchins has also been observed [4].

The genome of the purple sea urchin *Strongylocentrotus purpuratus* was sequenced as the first in echinoderm [5], which enabled great progress of genetic and genomic studies in sea urchin and its closely related species such as sea cucumber. However, the whole genome assembly of *S*. *purpuratus* remains in the stage of several thousand scaffolds and chromosome-level assembly is still not available, which prohibited its use in genetics studies such as integration with quantitative trait locus (QTL) and gene mapping analysis, and comparative genomics. The major limitation hindering the development of chromosome-level assembly is the lack of a high-density genetic map.

Construction of high-resolution linkage maps with large numbers of molecular markers is the prerequisite step for fine-scale QTL mapping and comparative genome analysis. A genetic linkage map of sea urchin using an interspecific cross between *S*. *nudus* and *S*. *intermedius* has been constructed with AFLP markers [6]. Although linkage analysis with the interspecific cross can be done using this AFLP map, it’s not adequate for QTL analysis due to the limited number of mapped markers and thereby the low resolution of the current linkage map.

With advances in high genotyping efficiency, automation, data quality, genome-wide coverage and analytical simplicity, SNPs have been widely used for genome-wide genetic analysis [7]. Based on next-generation sequencing technologies, RAD-Seq (restriction site associated DNA sequencing) method facilitates the rapid and cost-efficient discovery of large numbers of SNPs, and enables large-scale genotyping by sequencing hundreds to thousands of individuals [8, 9]. RAD-Seq data can be readily analyzed without any prior genome information, which makes the technique particularly applicable to non-model organisms. Recent applications of RAD-Seq have enabled linkage analysis and QTL mapping in model and non-model species, such as three-spine stickleback [10], Atlantic salmon [11], spotted gar [12], as well as scallop [13].

In this study, we report the construction of RAD-tag based high-density genetic maps with an interspecific cross produced by a female *S*. *nudus* and a male *S*. *intermedius*, and the initial mapping of QTL for body size. In addition, we presented its applicability to anchor the scaffolds of *S*. *purpuratus* genome assembly onto chromosomes, to facilitate QTL analysis and comparative genome analysis.

## Materials and Methods

### Resource families and samples collection

Animals used in this research were obtained from commercial sea urchin catches (Dalian Pacific Seafood Co., Ltd, China) and laboratory, therefore approval from any ethics committee or institutional review board was not necessary. F_1_ progenies were generated by a crossing between a single female *S*. *nudus* and a single male *S*. *intermedius*. A total of 100 offsprings were collected as samples for RAD-Seq and linkage mapping. The size of sea urchin test for each individual was measured in diameter for QTL analysis. Genomic DNA was isolated from all offsprings and two parents using the DNeasy Tissue kits (Qiagen) following the manufacturer’s protocol. The quantity and quality of the extracted DNA were determined using the NanoDrop ND-1000 spectrophotometer (NanoDrop Technologies). The quality of DNA was verified by agarose gel electrophoresis.

### RAD-tag sequencing

RAD libraries were prepared for the 100 offsprings and two parents following the methods similar to those previously described [14, 8]. Briefly, the restriction enzyme *SbfI* was used to digest the genomic DNA, and *SbfI* specific Illumina linkers each containing a unique barcode were ligated to each digested DNA sample. Individual samples were pooled into libraries. The quality and concentration were assessed using Bioanalyzer DNA 1000 kit (Agilent Technologies, Santa Clara, CA). The sequencing was performed using Illumina HiSeq 2000 for 50 bp single-end reads.

### SNP discovery and genotyping

Sequence reads from the Illumina sequencing were sorted according to the unique barcode tags and were quality-filtered using the FASTX-Toolkit (http://hannonlab.cshl.edu/fastx_toolkit/). Reads missing the restriction sites or with ambiguous barcodes and of low quality (score under 30) were discarded. The retained reads were sorted into loci and genotyped using Stacks software 0.9998 [15]. The likelihood-based SNP calling algorithm [10] implemented in Stacks evaluates each nucleotide position in every RAD-tag of all individuals, thereby differentiating true SNPs from sequencing errors. The parameters were set to a minimum stack depth of 30, a maximum of two mismatches allowed in a locus in an individual, and up to one mismatch between alleles.

### Linkage map construction

The genotype data were filtered based on the call rate of samples and markers before being used for linkage mapping. The sample call rate was >70% (i.e., at least 70% SNPs had genotypes called in each sample) and the marker call rate was >90% (i.e., a SNP was called in at least 90% of the samples). Markers heterozygous in just one parent were mapped using a pseudo-testcross strategy [16], and markers heterozygous in both parents were mapped as an F_2_ family [17].

Sex-specific maps were constructed for each parent. The genetic map was constructed using R/Onemap [18] and JoinMap4 [19]. The allocation of markers into linkage groups was conducted using R/OneMap. Linkage groups were formed using minimum LOD values of 8 and a maximum recombination fraction of 0.35. The JoinMap4 was used to order the markers in each linkage group with the regression mapping algorithm. By using the regression mapping algorithm and taking into account potential genotypic errors, it reduces the tendency to erroneously derive oversized linkage groups, which is often observed in construction of high-density genetic maps [20]. Map distances were calculated in centiMorgan (cM) using the Kosambi mapping function. Genetic linkage maps were graphically presented using the program MAPCHART 2.2 [21].

### Integration with S. purpuratus genome assembly

The genome assembly (v3.1) of purple sea urchin (*S*. *purpuratus)* was downloaded from SpBase (http://www.spbase.org/SpBase/index.php). The locations of mapped markers on the *S*. *purpuratus* genome assembly were determined via BLAST. The RAD-tags harboring SNP markers were used as queries to align with the genome assembly using the BLASTN with e-value cutoff of 1E-10. Assembly scaffolds were anchored onto linkage groups if at least two markers from the same linkage group were blasted onto the scaffolds. The order and orientation of these scaffolds within each linkage group was then determined based on the locations of markers relative to each other. The homologous relationships of linkage groups between the two sea urchin species were developed based on the anchoring of same assembly scaffolds.

### QTL analysis of body size

The QTL analysis was conducted using R/qtl [22]. Initially, one thousand permutations were run using Haley-Knott regression. The 5% significance level corresponded to an average LOD threshold of 3.8. The existence of single QTL was then tested using the R/qtl function, *scanone*, based on the interval mapping model. QTL intervals were then further examined for significance by determining the Bayesian credible interval. Genes in these credible intervals were identified based on the *S*. *purpuratus* genome assembly, and annotated by BLAST against public database.

## Results

### RAD-tag sequencing

**Fig 1:**
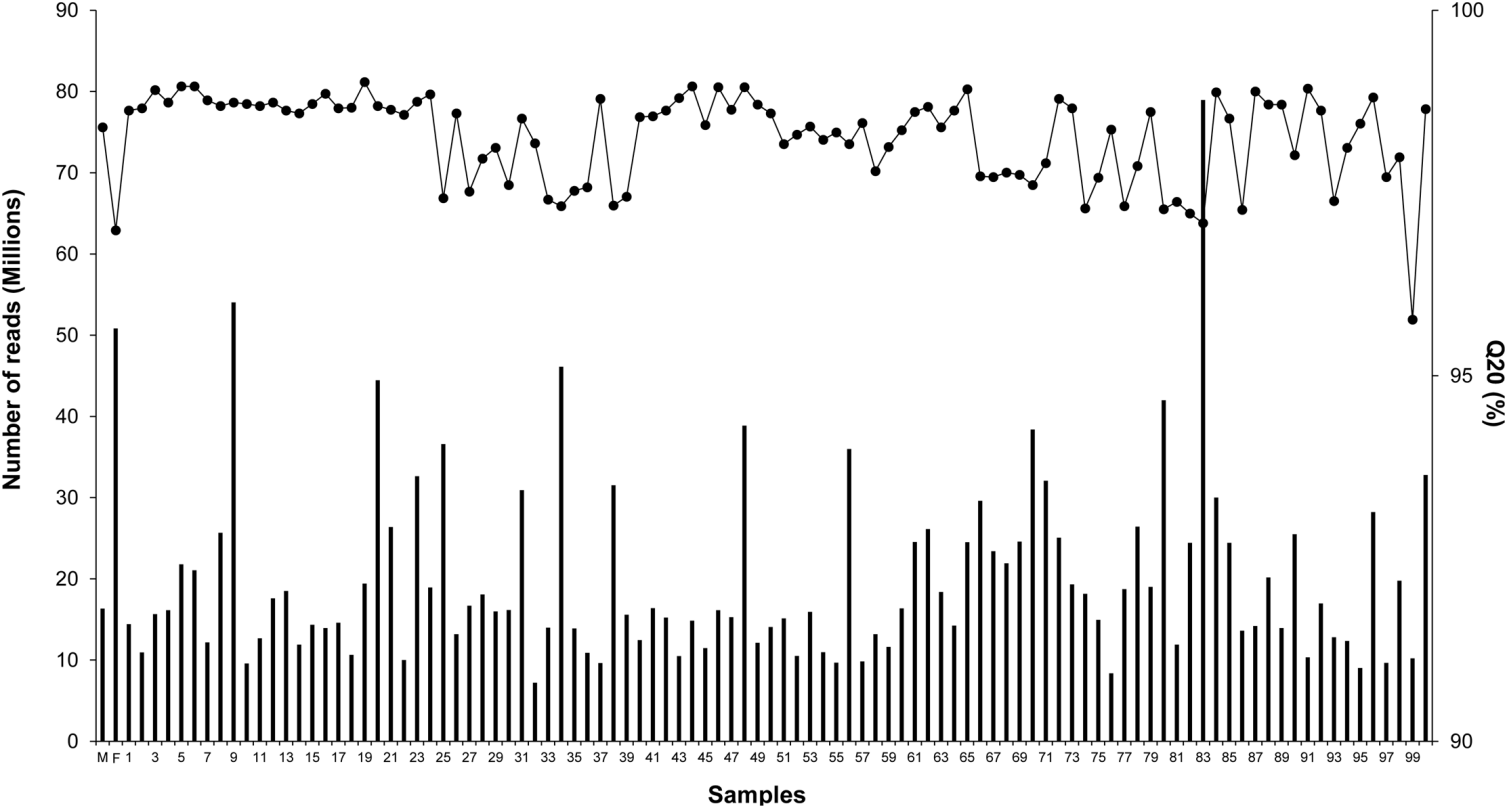
Generation of sequence tags with RAD-Seq.

RAD-tag sequencing of the 100 progenies and two parents yielded a total of 2,047,000,381 reads that passed Q20 filtering and were successfully processed for barcodes by Stacks program (Fig 1 and S1 Table). This corresponded to approximately a total of 87.6 Gb, accounting for over 107 X sequencing depth given the sea urchin genome size of 814 Mb [5]. A total of 50,842,078 reads were generated for the female, and 16,350,294 reads were generated for the male used for the crossing. On average, over 19 million clean reads were obtained for each progeny, accounting for an average of 1.04 X genome sequencing depth. The percentage of reads with quality score greater than Q20 were all above 95% with an average of 98.2%, suggesting that high quality sequencing reads were obtained in this work (Fig 1).

### Identification and genotyping of genetic markers

A total of 30,798 SNPs were successfully called with genotypes using the Stacks [10], including 21,108 markers segregated only in female, 9,602 segregated only in male, and only 88 SNPs were heterozygous in both parents. The dramatic differences in the number of informative markers between female and male was probably attributed to the differences in sequencing depth (Fig 1).

**Fig 2:**
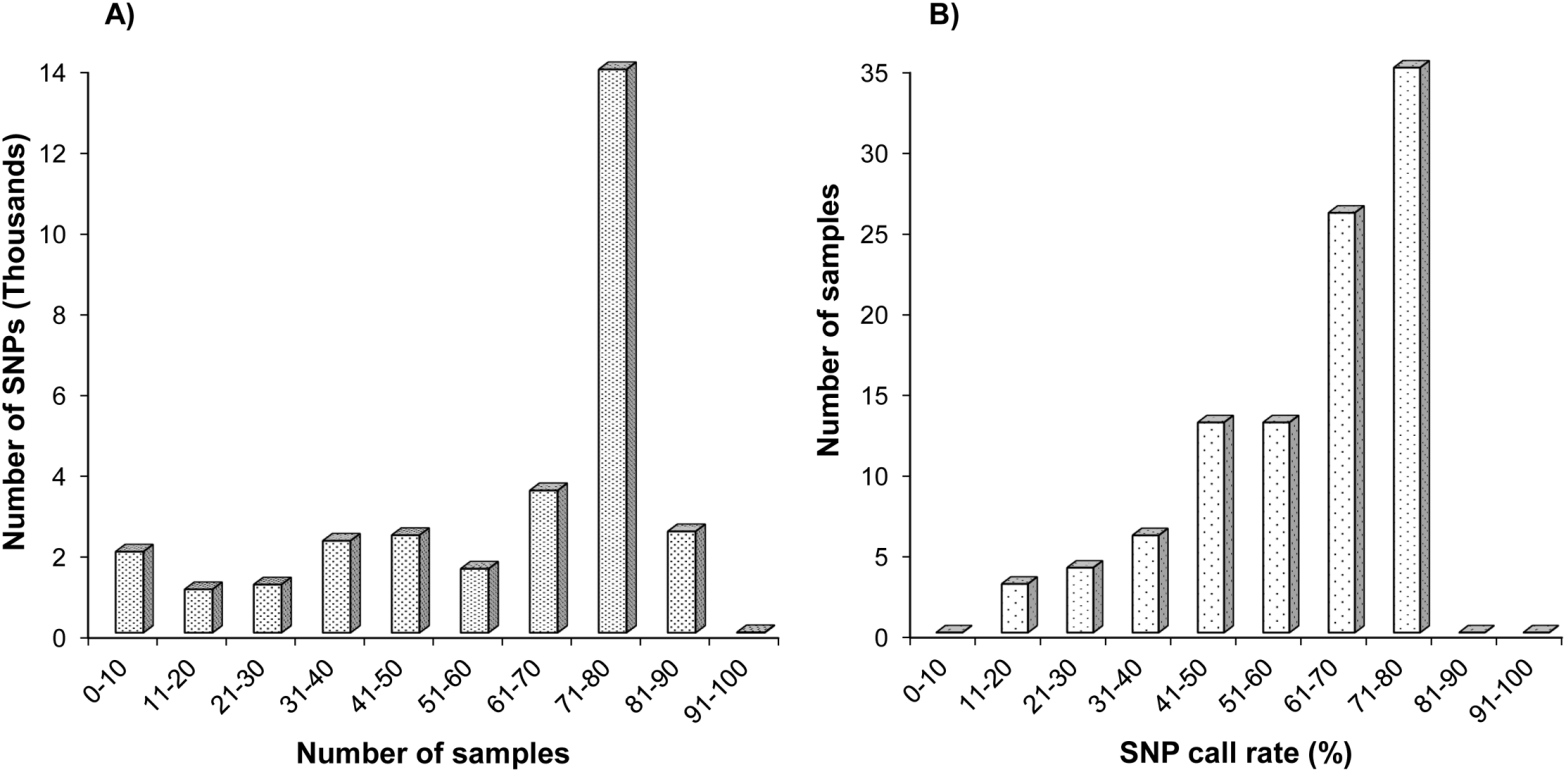
Genotyping and filtering of SNP markers for genetic mapping. A) Distribution of markers assessed by SNP call rate; B) Distribution of samples assessed by sample call rate.

Of the 30,798 SNPs, 16,507 SNPs were successfully genotyped in over 70 individuals. However, 9118 SNPs were genotyped in less than 50 individuals, including 2,042 SNPs that were only genotyped in less than 10 individuals, 1092 genotyped in 11–20 individuals, and 1212 genotyped in 21–30 individuals (Fig 2A). Of the 100 samples, 35 individuals had genotypes for over 70% of 30,798 SNPs, while a total of 13 individuals had genotypes for less than 40% SNPs (Fig 2B). High numbers of missing genotypes can be attributed to low sequencing depth and coverage in some of the progenies, with the number of sequencing reads ranging from 7,217,495 to 78,958,329 (Fig 1). In order to reduce effects of missing values and genotype errors, markers and samples were further filtered to keep only SNPs that were genotyped in at least 90 progenies (sample call rate >90%), and only the samples that had at least 70% SNPs being successfully genotyped. Lastly, we finalized a total of 9,616 SNPs and retained 75 individuals for genetic map construction.

### Genetic map construction

**Fig 3:**
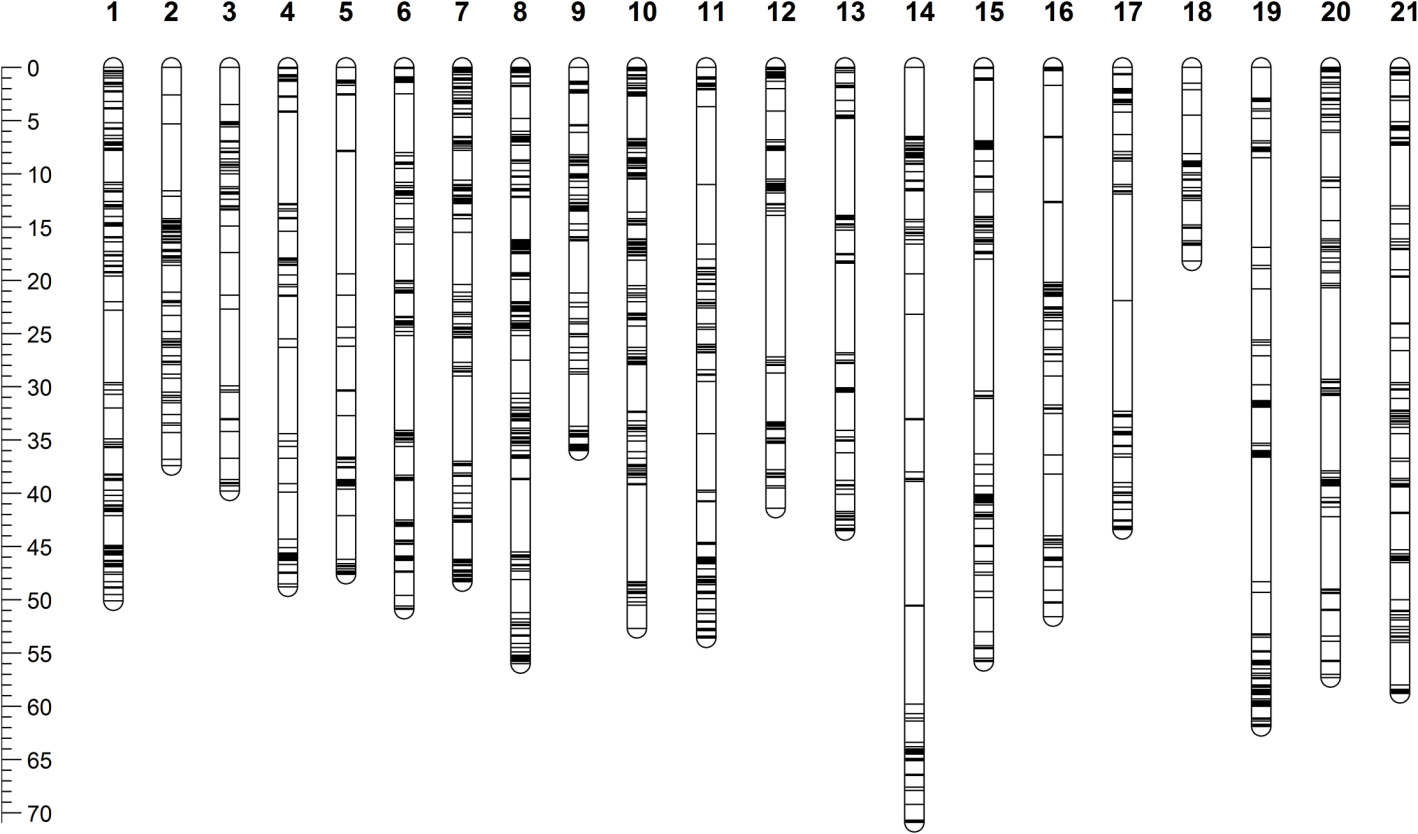
Genetic map of female *Strongylocentrotus nudus*.

**Fig 4:**
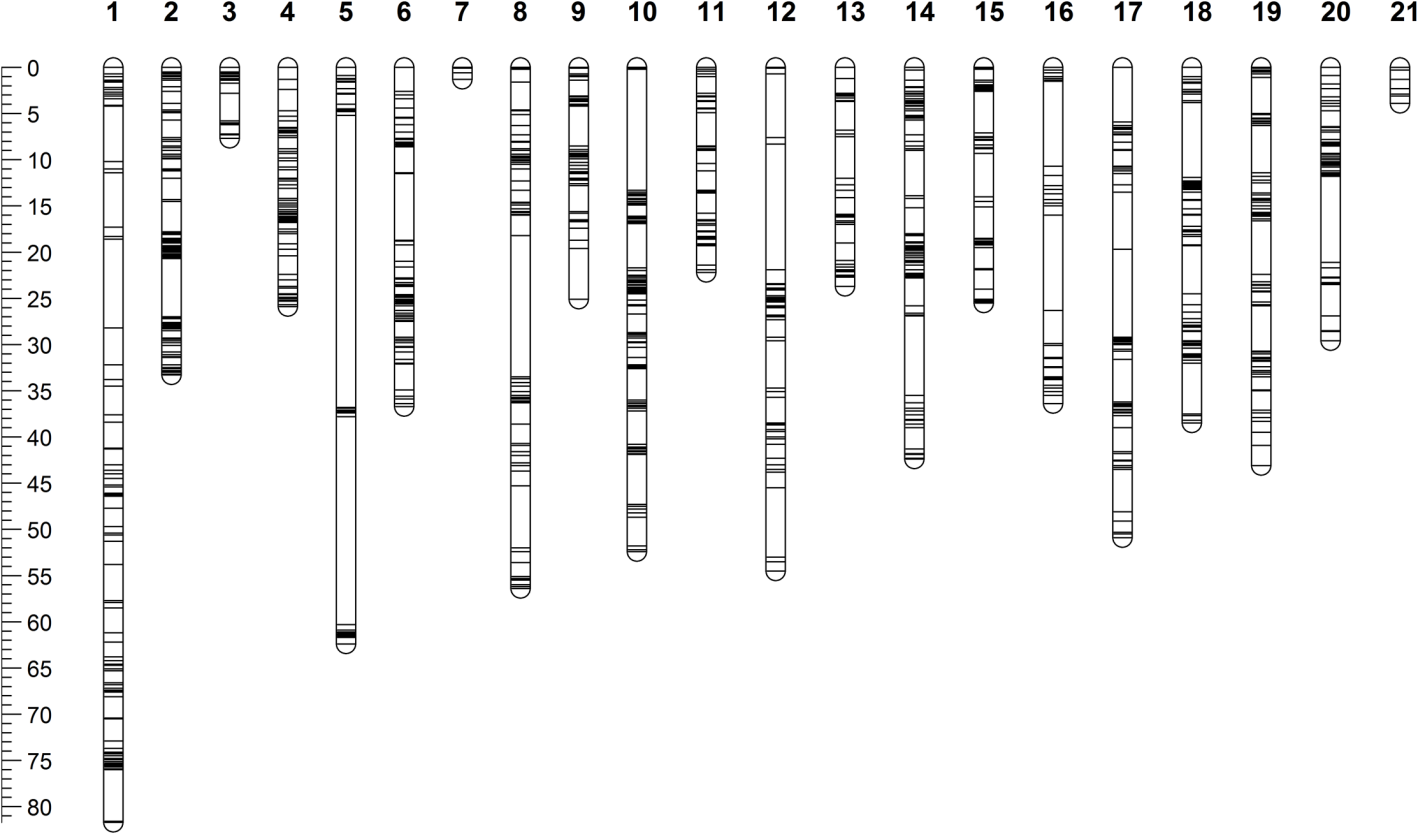
Genetic map of male *Strongylocentrotus intermedius*.

We constructed the female- and male-specific maps separately due to the large differences between the two species. Both female and male genetic maps were composed of 21 linkage groups (Figs 3 and 4), which is consistent with the chromosome number of haploid genome of *S*. *nudus* and *S*. *intermedius* [23, 24]. The homology of linkage groups between *S*. *nudus* and *S*. *intermedius* was determined based on the locations of their markers on the scaffolds of the *S*. *purpuratus* genome assembly. The one-to-one homologous relationships were clearly developed as listed in Table 1.

**Table 1:** Summary of genetic maps of *Strongylocentrotus nudus* and *S*. *intermedius*.

	Female map (*S*. *nudus*)			Male map (*S*. *intermedius*)		
Linkage group	No. of markers	Genetic length (cM)	Marker interval	No. of markers	Genetic length (cM)	Marker interval
**1**	**196**	**50.1**	**0.26**	**102**	**81.7**	**0.80**
**2**	**112**	**37.3**	**0.33**	**132**	**33.3**	**0.25**
**3**	**64**	**39.8**	**0.62**	**62**	**54.5**	**0.88**
**4**	**112**	**48.8**	**0.44**	**93**	**25.9**	**0.28**
**5**	**132**	**47.6**	**0.36**	**66**	**62.4**	**0.95**
**6**	**238**	**50.9**	**0.21**	**85**	**36.7**	**0.43**
**7**	**192**	**48.3**	**0.25**	**27**	**1.3**	**0.05**
**8**	**217**	**56**	**0.26**	**85**	**56.4**	**0.66**
**9**	**143**	**36**	**0.25**	**68**	**25.1**	**0.37**
**10**	**201**	**52.7**	**0.26**	**112**	**52.4**	**0.47**
**11**	**210**	**53.6**	**0.26**	**81**	**22.2**	**0.27**
**12**	**124**	**41.4**	**0.33**	**24**	**7.7**	**0.32**
**13**	**103**	**43.5**	**0.42**	**48**	**23.7**	**0.49**
**14**	**132**	**70.9**	**0.54**	**114**	**42.4**	**0.37**
**15**	**117**	**55.8**	**0.48**	**56**	**25.5**	**0.46**
**16**	**108**	**51.6**	**0.48**	**64**	**36.4**	**0.57**
**17**	**145**	**43.4**	**0.30**	**73**	**50.9**	**0.70**
**18**	**70**	**18.2**	**0.26**	**82**	**38.5**	**0.47**
**19**	**161**	**61.9**	**0.38**	**84**	**43.1**	**0.51**
**20**	**150**	**57.3**	**0.38**	**73**	**29.6**	**0.41**
**21**	**153**	**58.8**	**0.38**	**46**	**3.9**	**0.08**
**Total**	**3,080**	**1,023.9**	**0.33**	**1,577**	**753.6**	**0.48**

The female-specific map consisted of 3,080 SNP markers and spanned over 1,023.9 cM, with an average marker interval of 0.33 cM (Table 1). The male-specific map consisted of 1,577 SNP markers and spanned a total of 753.6 cM, with an average marker interval of 0.48 cM (Table 1). The detailed information of female and male genetic map was provided in S2 Table.

### Anchoring scaffolds of S. purpuratus genome assembly

Based on the linkage maps, we were able to anchor scaffolds of the *S*. *purpuratus* genome assembly (Table 2). The current version (v3.1) assembly of the *S*. *purpuratus* genome contained a total of 32,008 scaffolds (815.9 Mb), with average length of 25,491 bp and N50 length of 360,649 bp. A total of 1,591 scaffolds were anchored, with a total of 495.9 million bases, accounting for 60.8% of the *S*. *purpuratus* genome assembly. Of the anchored scaffolds, 453 scaffolds were anchored with two or more markers, which accounted for 31% of whole genome assembly. With two or more mapped markers, these scaffolds can be ordered and oriented based on the location of mapped markers. The information of the placement of scaffolds onto the linkage groups was provided in S3 Table.

**Table 2:** Summary of anchoring the *Strongylocentrotus purpuratus* genome assembly with linkage maps.

	Number of scaffolds	Scaffold size (Mb)	Percentage
**Whole genome assembly**	**32,008**	**815.9**	**100%**
**Total anchored scaffolds**	**1,591**	**495.9**	**60.8%**
**Scaffolds anchored with ≥2 SNPs**	**453**	**252.7**	**31.0%**

### QTL analysis

**Fig 5:**
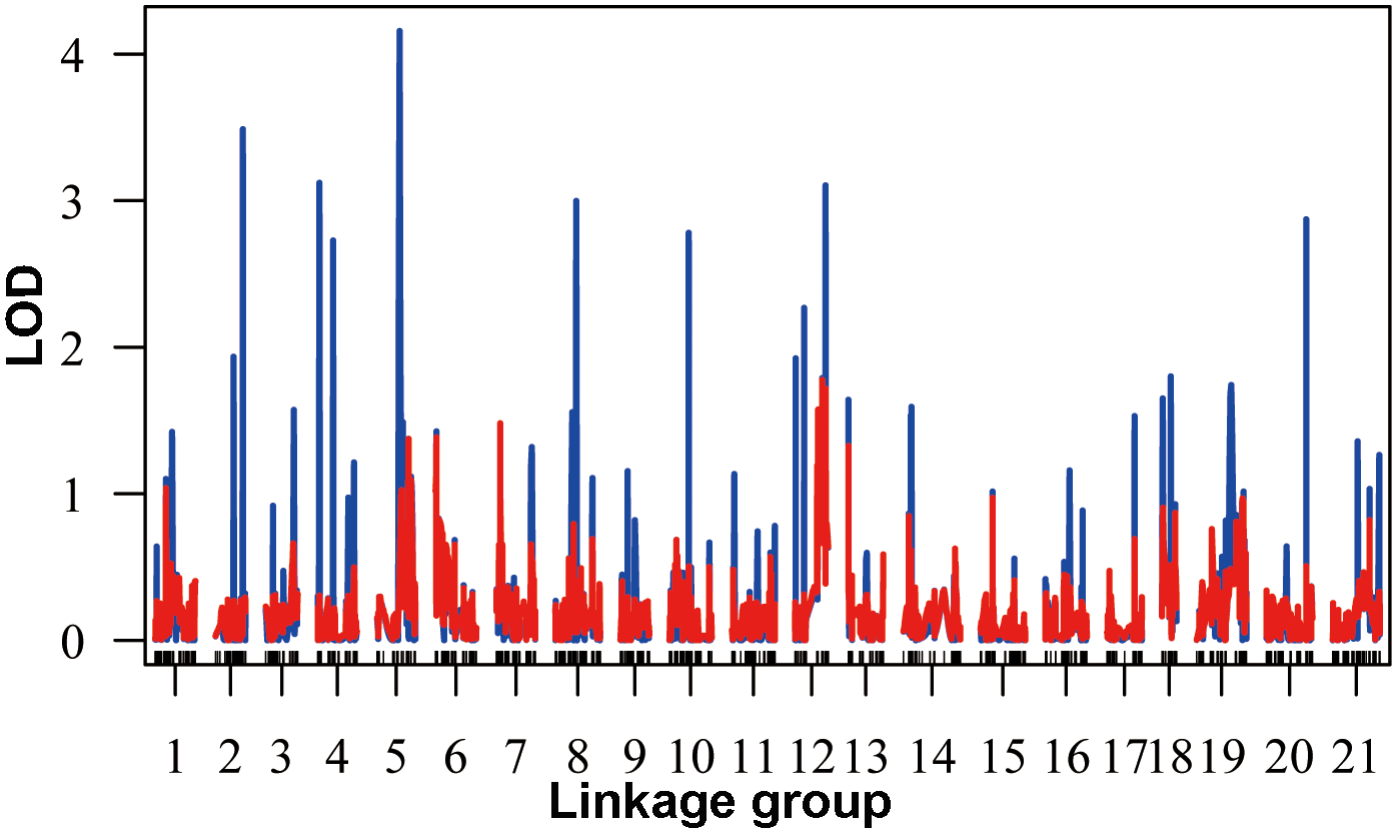
Genome wide distribution of LOD scores for body size. The red bars show the LOD generated from permutation tests (n = 5,000).

**Fig 6:**
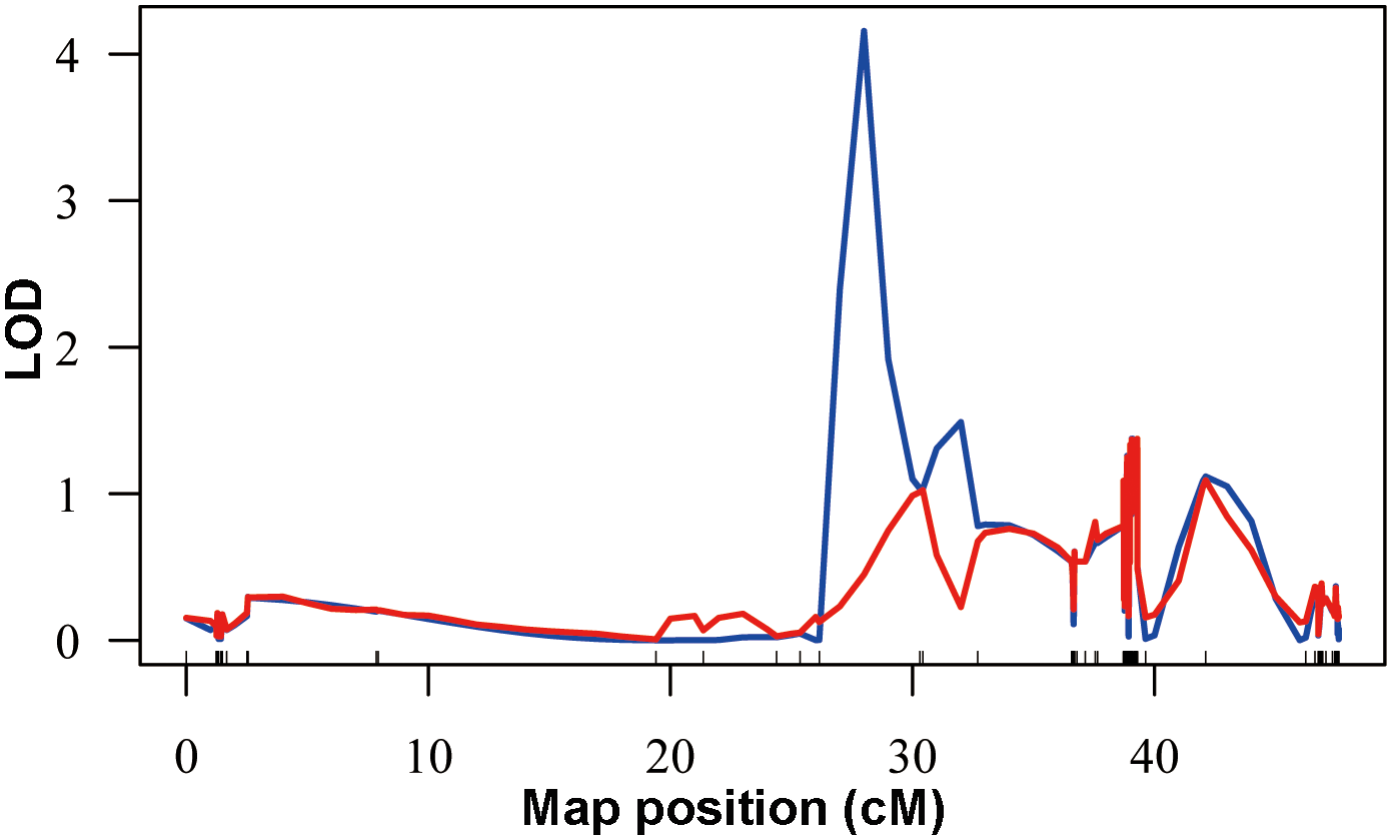
QTL plots for body size on linkage group 5.

A genome-wide scan resulted in the identification of one putative QTL for body size (Fig 5). The QTL with LOD score of 4.16 was identified on LG5 (Fig 6). The Bayesian 95% credible interval spans from 25.3 cM to 30.3 cM along LG5 (Fig 6). Through mapping with the genome assembly of *S*. *purpuratus*, two RAD-tag markers located at or near to the identified growth-related QTL were aligned with two genomic scaffolds, Scaffold421 and Scaffold561, respectively. Gene annotation of these two scaffolds revealed that a number of genes that might be involved in growth and development were identified from Scaffold561, including *Thyrotropin-releasing hormone receptor*, *Somatostatin receptor*, and *Kinesin-related proteins* (Table 3).

**Table 3:** Identification of growth-related genes from the QTL region based on the genome assembly of *Strongylocentrotus purpuratus*.

					Gene annotation		
Marker ID	LOD	Genetic position	Mapped genomic scaffolds	Genomic position of marker on scaffolds	Position	Accession	Gene name
**133078**	**0.02**	**24.4**	**-**	**-**	**-**	**-**	**-**
**124053**	**2.4**	**25.4**	**-**	**-**	**-**	**-**	**-**
**45359**	**4.2**	**26.2**	**-**	**-**	**-**	**-**	**-**
**31192**	**1.9**	**30.3**	**Scaffold421**	**80647–80687**	**1006–3017**	**XP_003726225**	***Proteasome activator complex subunit 4***
					**21179–29139**	**XP_783430**	***NFX1-type zinc finger-containing protein 1*-like**
					**37830–58904**	**XP_783383**	***NFX1-type zinc finger-containing protein 1*-like**
					**85609–93035**	**XP_003726682**	***NFX1-type zinc finger-containing protein 1*-like**
					**99281–128437**	**XP_003726678**	***NFX1-type zinc finger-containing protein 1*-like**
					**145853–148655**	**XP_003727478**	***Voltage-gated hydrogen channel 1*-like**
					**149713–153041**	**XP_003726679**	***Zinc finger protein 658B*-like**
					**157428–167565**	**XP_781443**	***Protein BANP*-like**
					**190574–196376**	**XP_003726680**	**Uncharacterized protein LOC100891444**
					**208034–217024**	**XP_799879**	**Uncharacterized protein LOC576126**
					**310244–321524**	**XP_003726681**	***Proteasome subunit alpha type-7*-like**
					**324710–331936**	**XP_794275**	***Dihydroorotate dehydrogenase* (quinone)**
					**333493–360838**	**XP_784540**	**Uncharacterized protein LOC579327**
					**388820–407045**	**XP_790027**	***Sodium-dependent noradrenaline transporter*-like**
					**474481–492033**	**XP_002741679**	***Formin*-*F*-like**
**325486**	**1.03**	**30.4**	**-**	**-**	**-**	**-**	**-**
**259227**	**0.78**	**32.7**	**Scaffold561**	**574803–574843**	**124–3647**	**XP_798750**	***WD repeat-containing protein 59*-like**
					**4529–12228**	**XP_003728075**	**Uncharacterized protein LOC100894008**
					**20308–51667**	**XP_001176245**	**Uncharacterized protein LOC752684**
					**87888–107446**	**XP_003727353**	**Uncharacterized protein LOC100889609**
					**110037–127664**	**XP_003731558**	**Uncharacterized protein LOC100890838**
					**142285–145735**	**XP_792763**	***Partitioning defective 6 homolog beta*-like**
					**157087–161272**	**XP_783156**	**Uncharacterized protein LOC577858**
					**162521–168812**	**XP_783224**	***NADH dehydrogenase flavoprotein 2***
					**174965–176710**	**XP_783290**	***Kelch-like protein 20*-like**
					**177674–201677**	**XP_001176034**	***Nodulation protein nolNO*-like**
					**220114–255066**	**XP_783602**	**Uncharacterized protein LOC578336**
					**343181–358582**	**XP_783839**	***Neuronal acetylcholine receptor subunit alpha-7*-like**
					**365339–386511**	**XP_003728617**	***Protein unc-13 homolog C*-like**
					**390211–396181**	**XP_003723955**	***Somatostatin receptor type 5*-like**
					**401480–443047**	**NP_999656**	***Kinesin-like protein KIF15***
					**458464–469028**	**XP_003727352**	***Thyrotropin-releasing hormone receptor*-like**
					**474600–477590**	**XP_783972**	***Thyrotropin-releasing hormone receptor*-like**
					**592344–612852**	**XP_006814515**	**Uncharacterized protein LOC102809399**
					**641275–655077**	**XP_003727356**	**Uncharacterized protein LOC100890028**

## Discussion

In this study, we report the construction of high-density genetic maps by genotyping F_1_ interspecific hybrids of a single family produced by a crossing between a female sea urchin *S*. *nudus* and a male *S*. *intermedius* using RAD-Seq technology. Based on polymorphisms present in these two wild individuals, we constructed a female meiotic map containing 3,080 markers for *S*. *nudus*, and a male meiotic map for *S*. *intermedius* which contains 1,577 markers. With these linkage maps, we were able to anchor a total of 1,591 scaffolds of the *S*. *purpuratus* genome assembly onto chromosomes, which accounted for 60.8% of the whole genome assembly. An initial QTL analysis resulted in the identification of one putative QTL for body size which spanned from 25.3 cM to 30.3 cM on linkage group 5. This study showed the efficiency of RAD-Seq to develop high-density genetic maps for species with no prior genomic information. The genetic maps provided in this study will be essential for QTL mapping and integration with genome assembly of related sea urchin species. The integration of the genetic map and genome sequence assembly would provide an unprecedented opportunity to conduct QTL analysis, comparative genomics, and population genetics studies.

The female- and male-specific maps were separately constructed. Construction of sex-averaged map was not attempted due to the large genetic differences existing between the two species (Table 1, Figs 3 and 4). Both female and male genetic maps were composed of 21 linkage groups, which is consistent with the chromosome number of haploid genome of *S*. *nudus* and *S*. *intermedius* [23, 24]. With the availability of the *S*. *purpuratus* genome assembly, we were able to clearly determine the homology of linkage groups between *S*. *nudus* and *S*. *intermedius* (Table 1) based on the locations of their markers on the genome scaffolds of the *S*. *purpuratus* genome assembly. It’s notable that only around a half number of markers were mapped onto the male map (*S*. *intermedius*) in comparison with that of the female map (*S*. *nudus)*. This could be due to the difference in diversity between the two individuals used in this study and the differences in the number of informative markers between female and male caused by sequencing depth.

The total map lengths were remarkably different between female and male, which were 1023.9 cM and 753.6 cM, respectively. The difference in recombination between two sexes has been observed in many organisms [25]. Although the molecular mechanisms related to the sex-specific recombination are still unclear, lower recombination ratio has been observed in the males of several fishes including rainbow trout, zebrafish, fugu and Japanese eel [26, 27, 28, 29], and one of the potential reasons is the reduced recombination around centromeres during male meiosis in teleosts [29]. In this study, the reductions of recombination were observed on both female and male maps, and there were no notable differences. Why the total female map length was longer than that of male in sea urchin warrants further investigations.

The genetic maps provided in this study enabled the integration of genetic maps with currently available sea urchin genome assembly. Because the genetic maps were constructed from polymorphisms present in RAD-tags, the sequences containing mapped markers can be associated with contigs/scaffolds in the genome assembly. Although the genetic divergences are present between the *S*. *purpuratus* and the species investigated in the present study, the alignment of the genetic map to the genome assembly still resulted in the anchoring of over 60% of the total genome scaffolds to chromosomal locations.

The QTL analysis of body size was able to be performed based on the constructed genetic map, and provided a putative QTL on linkage group 5 which spanned 5 cM. Although its lack of genome-wide significance due to the smaller individuals used (n = 75), the permutation test indicated that it’s associated with the body size more strongly than the expectation by chance (Fig 6). Due to the lack of the genome sequences of the investigated species, the *S*. *purpuratus* genome assembly was used for further investigation of the identified growth-related QTL region. Interestingly, several growth-related genes were identified (Table 3). Although chromosome rearrangements could occur between the *S*. *nudus* (whose map used for QTL analysis) and *S*. *purpuratus* during evolution, the identified growth-related genes deserve future studies to validate using other different families with large numbers of individuals.

## Conclusions

Using RAD-Seq, we were able to rapidly identify and genotype SNPs in an interspecific cross of sea urchin, allowing for construction of a high-density genetic map, with one marker approximately every 0.33–0.48 cM. Based on this genetic map, we anchored over 60% of the current *S*. *purpurtus* genome assembly, and identified a putative QTL region underlying body size, which was located on LG5, spanning from 25.3 cM to 30.3 cM. The integration of genetic maps with the *S*. *purpuratus* genome assembly enabled the investigation of the genome sequences within QTL regions to identify several growth-related genes. The genetic maps provided in this study will be essential for QTL mapping, and comparative genome analysis.

## Supporting Information

S1 Table Summary of RAD sequencing. (XLSX)

S2 Table Detailed information of female and male genetic maps. (XLSX)

S3 Table Scaffolding information of sea urchin genome assembly using the genetic maps presented in this study. (XLSX)
